# Upper limb kinematics after cervical spinal cord injury: a review

**DOI:** 10.1186/1743-0003-12-9

**Published:** 2015-01-30

**Authors:** Sébastien Mateo, Agnès Roby-Brami, Karen T Reilly, Yves Rossetti, Christian Collet, Gilles Rode

**Affiliations:** Université de Lyon, Université Lyon 1, INSERM U1028; CNRS UMR5292; Lyon Neuroscience Research Center, ImpAct Team, F-69676 Lyon, France; Hospices Civils de Lyon, Hôpital Henry Gabrielle, Mouvement et Handicap, F-69000 Lyon, France; Université de Lyon, Université Lyon 1, Centre de Recherche et d’Innovation sur le Sport, EA 647, Performance Motrice, Mentale et du Matériel, F-69621 Villeurbanne, France; Université de Paris, Université Paris 6, UPMC, Institut des systèmes intelligents et de robotique, CNRS UMR 7222, équipe Agathe INSERM U 1150, F-75006 Paris, France; Service de Médecine Physique et Réadaptation, Hôpital Henry Gabrielle, 20 route de Vourles, F-69230 Saint Genis Laval, France

**Keywords:** Tetraplegia, SCI, Upper limb, Reaching, Reach-to-grasp, Kinematic

## Abstract

Although a number of upper limb kinematic studies have been conducted, no review actually addresses the key-features of open-chain upper limb movements after cervical spinal cord injury (SCI). The aim of this literature review is to provide a clear understanding of motor control and kinematic changes during open-chain upper limb reaching, reach-to-grasp, overhead movements, and fast elbow flexion movements after tetraplegia. Using data from MEDLINE between 1966 and December 2014, we examined temporal and spatial kinematic measures and when available electromyographic recordings. We included fifteen control case and three series case studies with a total of 164 SCI participants and 131 healthy control participants. SCI participants efficiently performed a broad range of tasks with their upper limb and movements were planned and executed with strong kinematic invariants like movement endpoint accuracy and minimal cost. Our review revealed that elbow extension without *triceps brachii* relies on increased scapulothoracic and glenohumeral movements providing a dynamic coupling between shoulder and elbow. Furthermore, contrary to normal grasping patterns where grasping is prepared during the transport phase, reaching and grasping are performed successively after SCI. The prolonged transport phase ensures correct hand placement while the grasping relies on wrist extension eliciting either whole hand or lateral grip. One of the main kinematic characteristics observed after tetraplegia is motor slowing attested by increased movement time. This could be caused by (i) decreased strength, (ii) *triceps brachii* paralysis which disrupts normal agonist–antagonist co-contractions, (iii) accuracy preservation at movement endpoint, and/or (iv) grasping relying on tenodesis. Another feature is a reduction of maximal superior reaching during overhead movements which could be caused by i) strength deficit in agonist muscles like *pectoralis major*, ii) strength deficit in proximal synergic muscles responsible for scapulothoracic and glenohumeral joint stability, iii) strength deficit in distal synergic muscles preventing the maintenance of elbow extension by shoulder elbow dynamic coupling, iv) shoulder joint ankyloses, and/or v) shoulder pain. Further studies on open chain movements are needed to identify the contribution of each of these factors in order to tailor upper limb rehabilitation programs for SCI individuals.

Cervical spinal cord injury (SCI) leads to extensive sensorimotor deficits affecting both somatic (e.g. upper and lower extremity, trunk) and vegetative functions below the injury level [1]. A C5 SCI preserves innervation of shoulder and elbow flexors while C6 injuries spare wrist extensors and C7 injuries spare elbow extensors in addition (see Table 1). Thus, from a functional perspective, C5 and C6 injuries impair active elbow extension against gravity while C5 to C7 injuries prevent active grasping [2]. Fortunately, when wrist extension is preserved (i.e. injury at C6 or below), tenodesis can replace active grasp by passive whole hand and lateral grips. During wrist extension tenodesis causes passive tendon shortening of *flexor digitorum superficialis and profundus*, leading to passive finger-to-palm flexion, and of *flexor pollicis longus*, leading to thumb-to-index lateral face adduction [3].

**Table 1:** **Upper limb muscles function, innervation** [4] **and the detail of the consequences of spinal cord injury level on muscle innervation**

			Innervation		SCI level					
Joint	Muscles	Function	Nerve	Roots	C4	C5	C6	C7	C8	T1
Shoulder scapulo- thoracic	Serratus anterior	Protraction & upward rotation	Long thoracic	C5 C6 C7	**-**	**±**	**±**	**±**	**+**	**+**
	Trapezius upper part	Elevation	Accessory spinal	XI	**+**	**+**	**+**	**+**	**+**	**+**
	Trapezius middle part	Retraction			**+**	**+**	**+**	**+**	**+**	**+**
	Trapezius lower part	Downward rotation			**+**	**+**	**+**	**+**	**+**	**+**
	Pectoralis minor	Depression & anterior tipping	Medial pectoral	C8 T1	**-**	**-**	**-**	**-**	**±**	**±**
Shoulder gleno- humeral	Deltoïd anterior part & Coracobrachialis	Flexion	Axillary	C5 C6	**-**	**±**	**±**	**+**	**+**	**+**
	Deltoïd medius part	Abduction			**-**	**±**	**±**	**+**	**+**	**+**
	Deltoïd posterior part	Extension			**-**	**±**	**±**	**+**	**+**	**+**
	Pectoralis major upper part	Flexion/Adduction/Medial rotation	Lateral pectoral	C5 C6 C7	**-**	**±**	**±**	**±**	**+**	**+**
	Pectoralis major middle & lower parts	Flexion/Adduction/Medial rotation	Medial pectoral	C8 T1	**-**	**-**	**-**	**-**	**±**	**±**
	Lattissimus dorsi	Extension/Adduction/Medial rotation	Thoracodorsal	C6 C7 C8	**-**	**-**	**±**	**±**	**±**	**+**
	Teres major	Extension/Adduction/Medial rotation	Subscapularis	C5 C6 C7	**-**	**±**	**±**	**±**	**+**	**+**
	Subscapularis	Medial rotation			**-**	**±**	**±**	**±**	**+**	**+**
	Supraspinatus	Abduction			**-**	**±**	**±**	**±**	**+**	**+**
	Infraspinatus	Lateral rotation			**-**	**±**	**±**	**±**	**+**	**+**
	Teres minor	Lateral rotation	Axillary	C5 C6	**-**	**±**	**±**	**+**	**+**	**+**
Elbow	Biceps brachii	Flexion	Musculo-cutaneous	C5 C6	**-**	**±**	**±**	**+**	**+**	**+**
	Brachialis	Flexion			**-**	**±**	**±**	**+**	**+**	**+**
	Brachioradialis	Flexion			**-**	**±**	**±**	**+**	**+**	**+**
	Triceps brachii	Extension	Radial	C7 C8 T1	**-**	**-**	**-**	**±**	**±**	**±**
Wrist	Extensor carpi radialis longus & brevis	Extension	Radial	C6 C7 C8	**-**	**-**	**±**	**±**	**±**	**+**
	Extensor carpi ulnaris	Extension		C7 C8	**-**	**-**	**-**	**±**	**±**	**+**
	Flexor carpi radialis	Flexion	Median	C6 C7	**-**	**-**	**±**	**±**	**+**	**+**
	Flexor carpi ulnaris	Flexion	Ulnar	C7 C8	**-**	**-**	**-**	**±**	**±**	**+**
Fingers & thumb	Flexor digitorum superficialis	Flexion	Median	C7 C8 T1	**-**	**-**	**-**	**±**	**±**	**±**
	Flexor digitorum profundus	Flexion	Median & ulnar	C8 T1	**-**	**-**	**-**	**-**	**±**	**±**
	Extensor digitorum	Extension	Radial	C6 C7 C8	**-**	**-**	**±**	**±**	**±**	**+**
	Flexor pollicis longus & brevis	Flexion	Median	C8 T1	**-**	**-**	**-**	**-**	**±**	**±**
	Extensor pollicis longus & brevis	Extension	Radial	C7 C8	**-**	**-**	**-**	**±**	**±**	**+**
	Abductor pollicis longus	Abduction			**-**	**-**	**-**	**±**	**±**	**+**
	Abductor pollicis brevis	Abduction	Median	C8 T1	**-**	**-**	**-**	**-**	**±**	**±**
	Opponens pollicis	Opposition			**-**	**-**	**-**	**-**	**±**	**±**
	Adductor pollicis and intrinsic^1^	adduction &^2^	Ulnar	C8 T1	**-**	**-**	**-**	**-**	**±**	**±**
	Abductor digitorum minimi	Abduction	Ulnar	T1	**-**	**-**	**-**	**-**	**-**	**±**

^1^Intrinsic muscles are lumbricals, palmar and dorsal interossei; ^2^Functions of previous muscles are flexion of metacarpophalangeal and extension of both proximal and distal interphalangeal joints; adduction and abduction of fingers.

*Abbreviation:* *SCI* Spinal Cord Injury.

Muscle innervation depending on level of spinal cord injury was set as minus for non-innervated muscles, plus or minus for partially innervated muscles and plus for normally innervated muscles.

Autonomy after tetraplegia is based upon upper limb movements and is achieved by both re-learning open-chain movements like grasping, and learning novel closed-chain movements like manual wheelchair propulsion or sitting pivot transfer. This is achieved by rehabilitation [1, 2] and can be complemented by surgical tendon transfer which involves transferring a tendon from a spared muscle (i.e. one with a score above 4/5 at manual muscle testing) to that of a paralyzed muscle [5, 6]. If *triceps brachii* is paralyzed, the aim of the surgery is to restore active elbow extension, otherwise its aim is to restore active grasping [7–10].

Clinical assessments are typically used to document upper limb function as well as rehabilitation- or surgery- related improvements [11], but these tests are often subjective and less sensitive than kinematic recordings [12, 13]. Thus, to better characterize upper limb movements, kinematic tests have been developed based on reach-to-grasp [14, 15], drawing lines [16], overhead movements like shoulder flexion and abduction [17], wheelchair propulsion [18], or sitting pivot transfer [19, 20]. Kinematics have also been used to demonstrate intervention-related improvements in upper limb movements after C6 tetraplegia with [21] and without [22] tendon transfer.

Open-chain movements can be programmed and performed in different ways [23]. By examining these movements (that have to be re-learned after injury), SCI provides the opportunity to test theories of motor control [17, 24–28], as individuals with SCI should be able to plan movements despite having fewer degrees of freedom to execute them. Although a number of upper limb kinematic studies have been conducted, to date there are no reviews dedicated to addressing the key-features of open-chain movements after cervical SCI. The aim of this paper is to provide such a review, with particular emphasis on the modified kinematics of upper limb movements after tetraplegia.

## Methods

We consulted the U.S. National Library of Medicine® (MEDLINE) between 1966 and December 2014. We included full texts from peer-reviewed journals describing kinematics of upper limb movements in alive individuals with complete motor tetraplegia (i.e. AIS scores A and B). We included series case studies of open-chain upper limb movements without extra-load and excluded studies on human cadavers, single cases, and closed-chain upper limb movements.

We examined temporal kinematic parameters including movement time (MT), peak velocity (PV), time to peak velocity (TPV), peaks of acceleration and deceleration, number of PV, acceleration and deceleration peaks. We examined spatial parameters such as trajectory, hand height, joint motion, endpoint accuracy, displacement errors, and joint coordination. We also considered kinematic characteristics after surgery for restoration of elbow extension, and when available, characteristics of physiological recordings e.g. electromyographic activity.

## Results

### Included articles

We found 24 articles in the database and identified one additional study after reading the references of the 24 articles [29]. We thus examined 25 articles and rejected 7 of these 25 since they did not fulfill at least one of the inclusion criteria: incomplete lesions (AIS D) [16]; single case-study [21, 22]; no kinematic recordings [30–32]; human cadavers [33]. The 18 included studies addressed modifications in upper limb motor control during (i) reaching [3, 24–27, 34–36], (ii) reach-to-grasp [3, 14, 15, 29, 35, 37] and (iii) overhead movements such as shoulder flexion or abduction [17, 38–41]. One study focused on kinematics during fast elbow flexion [28].

### Participants

**Figure 1:**
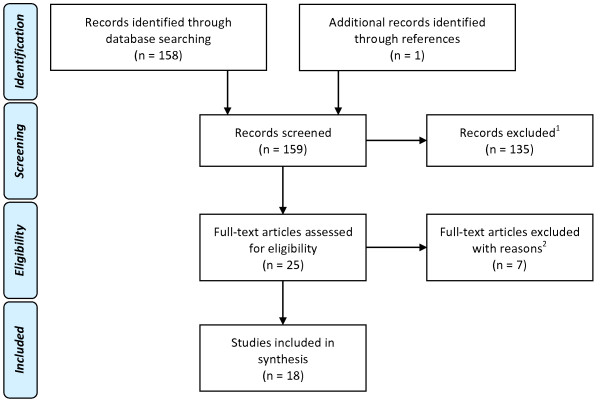
**Flow Diagram of the review process according to PRISMA guidelines** **[**[42]**].** ^1^Records were excluded because they studied upper limb in closed chain movements during weight relief transfer or wheelchair propulsion. ^2^Records were excluded since they did not fulfill at least one of the inclusion criteria: incomplete lesions (AIS D) [16]; single case-study [21, 22]; no kinematic recordings [30–32]; human cadavers [33].

The selected studies included a total of 164 SCI participants with complete motor deficit after injury and 131 healthy controls. Injury level was between C4 and C8. Only one study included high thoracic SCI between T1 and T4 [34] (see Figure 1 and Table 2).

**Table 2:** **Participant and study characteristics**

Authors	Study	SCI level	Patient number	Mean age (years)	Mean delay since injury (months)	Tasks	Kinematic device	EMG recording	Table height	Velocity
				***(range or SD)***	***(range or SD)***					
Acosta et al.	CC	C5	2	48 *(SD 2.6)*	NA	Overhead^i^	Optotrak™	No	No table	Comfortable
		C6	3							
Cacho et al.	CC	C6	11	30.5 *(SD 6.5)*	70.9 *(SD 44.0)*	Grasping^1^	Qualisys™	No	Elbow	Comfortable
		C7	9							
Gronley et al.	SC	C6	15	33 *(22 to 44)*	108 *(36 to 252)*	Overhead^ii^	VICON™	Inv	No table	Comfortable
Hoffmann et al.	SC	C6	9	28.7 *(24 to 34)*	30.5 *(7 to 120)*	Grasping^1^	Fastrak™	No	Elbow	Comfortable
		C7	2							
Hoffmann et al.	CC	C6	8	30.4 *(24 to 43)*	25.4 *(5 to 56)*	Reaching^a^	Fastrak™	No	Elbow	Comfortable
		C7	7	28.8 *(24 to 36)*	70.2 *(7 to 262)*					
Jacquier-Bret et al.	CC	C6	5	39.6 *(SD 9.7)*	NA	Grasping^2^	Flocks of Birds™	Surf	Elbow	Comfortable
Koshland et al.	CC	C6	5	NA *(25 to 37)*	*NA (132 to 216)*	Reaching^b^	Videotape recording	Surf	Elbow	Quick
Laffont et al.	SC	C6	4	29 *(26 to 34)*	10 *(5 to 19)*	Reaching^c,d^	Fastrak™	No	Elbow	Comfortable
						Grasping^1^				
Maksimovic et al.	CC	C4	2	33.1 *(3 to 60)*	68.7 *(12 to 144)*	Overhead^iii^	Goniometer	No	No table	Comfortable
		C5	2							
		C6	5							
		C7	7							
Mateo et al.	CC	C6	4	27.5 *(SD 8.3)*	68 *(6 to 216)*	Reaching^c^	VICON™	No	Elbow	Comfortable
						Grasping^3,4^				
Nunome et al.	CC	C7	5	32.6 (SD 2.4)	NA *(108 to 252)*	Basketball throw	Videotape recording	No	No table	Comfortable
		C8	1							
Popovic et Popovic	CC	C6	6	NA	NA	Reaching^d^	Goniometer	No	Shoulder	Comfortable
Robinson, Barton et al.	CC	C5	2	42.8 *(SD 12.1)*	183.3 *(SD 126.9)*	Reaching^e^	Qualisys™	No	No table	Comfortable
		C6	8							
		C7	1							
Robinson, Hayes et al.	CC	C6	5	39 *(SD 9)*	211.2 *(SD 60.4)*	Reaching^f^	Qualisys™	No	Elbow	Quick
Reft et hasan	CC	C7	1	25.8 *(23 to 35)*	*NA (36 to 92 )*	Reaching^g^	Selspot™	No	No table	Quick
		T1 and T2	2							
		T4	2							
Remy-Neris et al.	CC	C6	5	27.0 *(SD 6.0)*	Chronic SCI	Overhead^iv^	VICON™	No	No table	Comfortable
Reyes-Guzman et al.	CC	C6	8	33.6 *(SD 13.0)*	8.5 *(SD 2.2)*	Grasping^5^	Codamotion™	No	Elbow	Comfortable
		C7	8	28.8 *(SD 9.8)*	7.5 *(SD 1.9)*					
Wierzbicka et al.	CC	C5	7	40 *(21 to 64)*	149.3 *(3.6 to 432)*	Elbow flexion	Homemade	Surf	Shoulder	Quick

Reaching: ^a^with a pointer fixed at dorsum of the hand; ^b^with arm rolling on table and wrist splinted; ^c^target on the table; ^d^object non specified; ^e^targets at 5 locations of reachable space; ^f^hand rolling over table with frictionless system; ^g^2 targets set at ipsilateral shoulder level.

Grasping: ^1^a cone; ^2^a low mass cubic object; ^3^apple; ^4^vertical floppy disk; ^5^a glass.

Overhead movements: ^i^Shoulder flexion, abduction in frontal plane and in scapula plane with elbow extended; ^ii^Shoulder flexion and abduction and 4 ADL (consisting in drinking from an empty cup, flipping a lightswitch, hair combing, and reaching for the perineum.); ^iii^Shoulder flexion and abduction; ^iv^Shoulder flexion with elbow extended or hand to nape of the neck.

*Abbreviations:* *SCI* Spinal cord injury, *NA* not available, *SC* series case, *CC* control case, *ADL* activity of daily life, *Inv* invasive, *Surf* surface.

### Apparatus

In all studies, the SCI participants sat in their wheelchair while control participants, when included, sat on a standard chair. One study provided no information about trunk stabilization [27], but in all other studies a strap stabilized the chest to the back of the seat except in two studies [34, 37] where the trunk had no restriction for anterior motion. Systems used to record 3D motion were electromagnetic, electro-goniometric, optoelectronic, or videographic (see Table 2).

### Reaching movements

#### Temporal kinematics

*After C5 to C7 SCI* MT increased [3, 25–27, 35], PV decreased [3, 24, 26, 27, 34, 35], but TPV was similar to that of control participants [3, 26, 27]. Compared with control participants, PV was approximately 30% lower and MT more than 40% slower (see Table 3). *After C6 and C7 SCI* the velocity profiles of the hand and fingers remained bell-shaped [3, 24, 25, 35]. *After C6 SCI* variability in PV increased [35], the magnitude of the maximum acceleration and deceleration peaks was reduced and there were multiple acceleration peaks [25] (see Tables 3 and 4).

**Table 3:** **Review of movement time and velocity peak in SCI and control participants**

Authors	Tasks	Distance (cm)	PV (m/s)			MT (s)		
			SCI	Control	SCI/control (%)	SCI	Control	SCI/control (%)
Hoffmann et al.	R	Arm length	0.87 ± 0.04	1.00 ± 0.04	13.00	NA	NA	NA
Koshland et al.	R	20	0.60^a^	1.30	53.85	NA	NA	NA
Laffont et al.	R	Low	0.83 ± 0.25	1.08 ± 0.31	23.15	NA	NA	NA
		High	1.10 ± 0.31	1.47 ± 0.25	25.17			
Mateo et al.	R	28^b^	0.66 ± 0.17	0.94 ± 0.22	29.79	0.66 ± 0.20	0.44 ± 0.14	50.00
			0.78 ± 0.19	1.15 ± 0.25	32.17	0.67 ± 0.24	0.47 ± 0.14	42.55
Popovic et Popovic	R	NA	NA	NA	NA	NA	NA	NA
Reft et Hasan	R	Far no support	0.35 ± 0.07	0.50 ± 0.09	30.10	NA	NA	NA
		Far support	0.32 ± 0.04	0.48 ± 0.07	33.19			
Robinson, Barton et al.	R	Arm length	NA^c^	NA^c^	NA	NA^c^	NA^c^	NA
Robinson, Hayes et al.	R	20	0.59	0.92	35.87	0.67	0.49	36.73
Cacho et al.	G	Arm length	0.79 ± 0.14	0.88 ± 0.14	10.23	1.53 ± 0.08	0.92 ± 0.16	66.3
Hoffmann et al.	G	Arm length	1.07 ± 0.05	1.22 ± 0.06	12.3	NA^d^	NA^d^	NA
Jacquier-Bret et al.	G	40	0.7^e^	0.9^e^	22.22	NA^c^	NA^c^	NA
Laffont et al.	G	Short	0.92 ± 0.25	1.08 ± 0.31	14.81	3.12 ± 0.85	1.81 ± 0.45	72.38
		Long	1.04 ± 0.28	1.19 ± 0.31	12.61	3.30 ± 1.08	1.82 ± 0.25	81.32
Mateo et al.	G	35	0.71 ± 0.13	0.80 ± 0.08	11.25	1.90 ± 0.60	0.77 ± 0.09	146.75
			0.67 ± 0.09	0.69 ± 0.08	2.90	1.64 ± 0.13	0.75 ± 0.12	118.67
Reyes-Guzman et al.	G	18	0.56 ± 0.26^f^	0.66 ± 0.09	15.15	2.57 ± 0.98^f^	1.04 ± 0.33	147.12
			0.67 ± 0.53^g^		-1.52	1.66 ± 1.07^g^		59.62
Acosta et al.	O	NA	NA	NA	NA	NA	NA	NA
Gronley et al.	O	NA	NA	NA	NA	NA	NA	NA
Maksimovic et al.	O	NA	NA	NA	NA	0.65 ± 0.17	0.45	44.44
Nunome et al.	O	2.16	4.26 ± 0.67^h^	5.45 ± 0.25^h^	21.83	NA	NA	NA
Remy-Neris et al.	O	NA	62 ± 22^*i*^	201 ± 41^*i*^	69.15	NA	NA	NA
			109 ± 39^*ii*^	241 ± 29^*ii*^	54.77			
Wierzbicka et al.	E	NA	NA	NA	50	twice control	NA	200

^a^For n = 4 SCI, only one exhibit 1.1 m/s velocity; ^b^Targets were placed centrally or laterally on the right; ^c^Non significant differences were reported without data available.

^d^Increase time between end of reaching and grasping in SCI participants; ^e^Value rounded extracted from figure; ^f^Value for C6 SCI participants; ^g^Value for C7 SCI participants; ^h^Vertical component of basketball after shooting; ^i^Maximal shoulder flexion and ^ii^maximal elbow flexion both expressed in degree per seconds in italics.

*Abbreviations:* *NA* Not Available, *P* Pointing, *G* Grasping, *O* Overhead, *E* Elbow flexion.

*MT* Movement Time, *PV* Velocity Peak; *cm* centimeter, *m/s* meter per seconds, *s* seconds.

**Table 4:** **Kinematic characteristics of reaching movements after tetraplegia**

Parameters		Main results	SCI motor level			References
			C5	C6	C7	
Movement time (MT)	T	Increased	**X**	**X**	**X**	[3, 25–27, 35]
Velocity Peak (PV)	T	Decreased	**X**	**X**	**X**	[3, 24, 26, 27, 34, 35]
		Variability increased		**X**		[35]
Acc & dec peaks	T	Reduced magnitude		**X**		[25]
Number of acc peaks	T	Multiple		**X**		[25]
Time to Velocity Peak (TPV)	T	Similar to control	**X**	**X**	**X**	[3, 26, 27]
Velocity profile^1^	T	Bell-shaped		**X**	**X**	[3, 24, 25, 35]
Velocity coordination	T	Shoulder velocity equal to half of elbow velocity		**X**		[36]
Trajectory^2^	S	Straight and smooth		**X**	**X**	[25, 35]
		Variability increased			**X**	[34]
Hand height	S	Not increased		**X**	**X**	[3, 24, 35]
Joint motion	S	Similar pattern of joint rotation		**X**	**X**	[24, 25]
		Increased of scapula movement		**X**		[35]
		Decreased of sup and med max reaching	**X**	**X**	**X**	[26]
		Decreased of acromion displacement		**X**	**X**	[34, 35]
Endpoint accuracy	S	Preserved		**X**	**X**	[3, 24, 25, 27]
Joint coordination^3^	S	Linear temporal relationship		**X**	**X**	[24, 35]
EMG activity	N	Activation of agonist without antagonist muscles		**X**		[25]
		Selective activation of shoulder muscles		**X**		[25]
		Prolonged muscle activation		**X**		[25]

^1^of hand and finger; ^2^of elbow, hand and finger; ^3^of shoulder and elbow.

*Abbreviations:* *T* Temporal kinematic parameter, *S* Spatial kinematic parameter, *N* Non kinematic parameter, *acc* acceleration, *dec* deceleration, *max* maximal, *sup* superior, *med* medial.

#### Spatial kinematics

*After C5 to C7 SCI* elbow, hand, and finger trajectories remained straight and smooth [25, 35]. Movement amplitude with the upper limb fully extended was reduced during maximal reaching in the superior and medial workspace [26]. *After C6 and C7 SCI* the pattern of joint rotation was similar to that of healthy participants [24, 25], with an increase in hand height when the target was placed on the table at elbow height [3, 24, 35], a decrease in acromion displacement [34, 35], and preserved endpoint accuracy [3, 24, 25, 27]. Shoulder and elbow joint motion followed a linear relationship attesting to the preservation of temporal coordination [24, 35]. *After C6 SCI* scapula movements increased [35] with more lateral scapula rotation during reaching in the lateral workspace than in healthy participants [24]. Scaling between shoulder and elbow rotation velocity was also preserved [36]. *After C7 SCI* finger trajectory variability increased [34] (see Table 4).

#### EMG activity

*After C6 SCI* agonist muscles were not activated in a reciprocal pattern as in healthy individuals. Instead, shoulder muscles were almost exclusively active [25, 43], and *pectoralis major* (a shoulder agonist) was active throughout the entire movement, whereas in controls it was active for only part of the movement [25] (see Table 4).

#### Effect of elbow extension restoration

*After tendon transfer in C5 to C7 SCI* a slight increase in shoulder flexion and maximal height reached in the superior and medial directions was reported, but these changes did not reach significance [26].

### Reach-to-grasp movements

#### Temporal kinematics

*After C6 and C7 SCI* MT increased [3, 14, 35, 37] and PV decreased [3, 14, 29, 35]. When compared with control participants, MT was nearly twice as long and PV was around 11% lower (see Table 3). Due to the longer MT, TPV was delayed [3, 14], but occurred earlier than in control participants when normalized to MT [37]. Several studies reported that in SCI participants there was an additional velocity peak between the go and return peaks in the reaching phase [29, 35, 37]. Interestingly, in one of these studies PV was negatively correlated with ASIA motor index [37]. *After C6 SCI* variability in PV increased [35] and the velocity profile was asymmetric, with a prolonged deceleration phase [3, 35]. During reaching before grasping, velocity of shoulder flexion, elbow extension, and wrist tangential velocity were stable (the latter was equal to 20% of its maximum) [15] (see Tables 3 and 5).

**Table 5:** **Kinematic characteristics of reach-to-grasping movements after tetraplegia**

Parameters		Main results	SCI motor level			References
			C5	C6	C7	
Movement time (MT)	T	Increased		**X**	**X**	[3, 14, 35, 37]
Velocity Peak (PV)	T	Decreased		**X**	**X**	[3, 14, 29, 35]
		Variability increased		**X**		[35]
		Negative correlation with ASIA score		**X**	**X**	[37]
Time to PV (TPV)	T	Delayed time to PV		**X**	**X**	[3, 14, 37]
Number of PV	T	Two peaks (reaching and return)		**X**	**X**	[29, 35]
		Additional PV (grasping)		**X**	**X**	[29, 35, 37]
Velocity	T	Stable^1^ before grasping		**X**		[15]
Velocity profile^2^	T	Asymmetric with a prolonged decelerative phase		**X**		[3, 35]
Trajectory^3^	S	Straight and smooth		**X**		[35]
Trajectory^4^	S	Reduced and less straight		**X**		[35]
Trajectory^5^	S	Increased curvature index		**X**	**X**	[37]
Trajectory^6^	S	Negative correlation with FIM		**X**	**X**	[37]
Hand height	S	Increased at the end of the reaching phase		**X**	**X**	[3, 29, 35]
Joint motion	S	Similar pattern of joint rotation		**X**	**X**	[14, 37]
		Increased of scapula movement		**X**		[15, 35]
		Increased wrist flexion/extension range of motion		**X**	**X**	[3, 14, 29]
		Wrist flexion during reaching		**X**	**X**	[3, 14, 15, 29]
		Wrist extension for grasping and manipulating		**X**	**X**	[3, 14, 15, 29]
		Increased of wrist extension for LG vs WHG		**X**		[3]
Joint coordination^7^	S	Temporal linear relationship		**X**	**X**	[14, 29, 35]
Joint coordination^8^	S	Temporal decoupling		**X**	**X**	[29]
EMG activity	N	Increased		**X**		[15]
		Activation of agonist without antagonist muscle		**X**		[15]

^1^for shoulder flexion, elbow extension and wrist tangential velocity; ^2^of hand and finger; ^3^of elbow, hand and finger; ^4^of acromion; ^5^of wrist; ^6^of index; ^7^of shoulder and elbow; ^8^of shoulder and wrist; ^a^2002.

*Abbreviations:* *T* Temporal kinematic parameter, *S* Spatial kinematic parameter, *N* Non kinematic parameter, *PV* Velocity Peak, *FIM* Functional Independence Measure, *LG* Lateral Grip, *WHG* Whole Hand Grip.

#### Spatial kinematics

*After C6-C7 SCI* the pattern of joint rotation was similar to that of control participants [14, 37], but hand height increased at the end of the reaching phase [3, 29, 35]. Index finger trajectory variability increased and was negatively correlated with the Functional Independence Measure score [37]. The range of motion at the wrist joint during reaching was greater than in control participants [3, 14, 29], with the wrist flexed during reaching [3, 14, 15, 29] and extended during grasping and object manipulation [3, 14, 15, 29]. Motion at the shoulder and elbow joints was linearly correlated, attesting to preserved temporal joint coordination [14, 29, 35], but motion at the shoulder and wrist joints was temporally desynchronized [29]. *After C6 SCI* scapula movements increased during reach to grasp [15, 35] while the acromion trajectory was shorter and less linear [35]. During tenodesis, wrist extension angle was greater for lateral than whole hand grip [3] (see Table 5).

#### EMG activity

*After C6 SCI* contraction of the agonists increased but contrary to healthy participants this increase was not paralleled by increased contraction in the antagonists [15] (see Table 5).

### Overhead movements

#### Temporal kinematic

*After C6 SCI* MT increased and shoulder velocity decreased [17]. *After C6 to C8 SCI* wrist flexion velocity decreased [40] while elbow velocity remained unchanged [17, 40]. PV was almost half that of control participants, and MT nearly one and a half times slower (see Tables 3 and 6).

**Table 6:** **Kinematic characteristics of overhead upper limb movements**

Parameters		Main results	SCI motor level				References
			C5	C6	C7	C8	
Movement time (MT)	T	Increased		**X**			[17]
Velocity	T	Decreased at shoulder joint		**X**			[17]
Velocity	T	No differences at elbow joint		**X**	**X**	**X**	[17, 40]
Velocity	T	Decreased for wrist flexion		**X**	**X**	**X**	[40]
Trajectory ^1^	S	Variability increased	**X**	**X**	**X**		[39]
Joint motion	S	Similar pattern of joint rotation	**X**	**X**	**X**		[39]
		Pattern of joint rotation variability increased	**X**	**X**	**X**		[38, 39]
		Decreased shoulder flexion and increased abduction	**X**	**X**	**X**	**X**	[17, 40, 41]
		Increased upward and forward shoulder motion			**X**	**X**	[40]
		Winging^2^ and adducted scapula during rest	**X**	**X**			[41]
		Decreased scapula lateral rotation during shoulder flexion	**X**	**X**			[41]
		Increased elbow flexion		**X**			[17]
Joint coordination ^3, 4^	S	Temporal linear relationship	**X**	**X**			[17, 41]
EMG activity	N	Decreased strength for shoulder rotator and elbow flexors		**X**			[28, 38]
		Increased contraction intensity for same task		**X**			[38]
Effect of elbow extension restoration	T	Reduced movement duration		**X**			[17]
	T	Increased shoulder joint velocity		**X**			[17]
	S	Increased shoulder flexion and decreased abduction		**X**			[17]
	S	Decreased elbow flexion		**X**			[17]

^1^of the hand; ^2^lower and medial part rotated outwards from the thorax ^3^between glenohumeral and scapulothoracic joints, ^4^between glenohumeral and elbow joints.

*Abbreviations:* *T* Temporal kinematic parameter, *S* Spatial kinematic parameter, *N* Non kinematic parameter.

#### Spatial kinematics

*After C5 to C7 SCI* participants presented less linear in hand trajectory and larger shoulder and elbow joint rotations than healthy controls [39]. *After C5 to C8 SCI* active shoulder flexion angle decreased while abduction angle increased compared with healthy controls [17, 40, 41]. After C5 and C6 SCI, the scapula was located more medially and exhibited ‘winging’ i.e. protrusion of the lower and medial parts from the thorax in the resting position. During shoulder flexion, there was less lateral rotation of the scapula for SCI individuals compared with controls [41]. *After C6 SCI* passive range of motion decreased during shoulder flexion (133°, SD = 9°) and abduction (133°, SD = 10°) [38] compared with healthy individuals (flexion = 156°, SD = 9° and abduction = 161°, SD = 11°) [44]. Movements of the glenohumeral joint were linearly related to both elbow and scapulothoracic joint movements attesting to preservation of temporal coupling [17, 41]. After C7 and C8 SCI, forward and upward displacement of the shoulder increased [40] (see Table 6).

#### EMG activity

*After C6 SCI* shoulder and elbow maximal isometric strength decreased [38]. Shoulder internal and external rotator strength decreased by 67% and 39% of control capacity, respectively [38], and contraction intensity increased [38] (see Table 6).

#### Effect of elbow extension restoration

Elbow extension restoration *after C6 SCI* altered kinematics towards patterns observed in control participants as it reduced prolonged MTs [17], and increased shoulder flexion angle, which in turn decreased shoulder abduction angle and scapula lateral rotation [17] (see Table 6).

### Effects of extensor impairment on elbow flexion

Only one study analyzed the accuracy of fast, single-joint elbow flexion of either 10°, 20° or 30° with and without a constant extensor torque provided by a manipulandum [28]. *After C5 SCI* the maximal isometric strength of elbow flexors was about 50% of controls (mean 46.3 Nm versus 78 Nm). Without elbow extensor torque, movements were smooth but slower than in controls, and there were more errors. In contrast, movement velocity increased and errors decreased when elbow extensor torque was provided.

### General discussion

Individuals with SCI can relearn open-chain movements despite reduced degrees of freedom and even though the kinematics of these movements differ from those of normal controls they preserve several kinematic invariants like movement endpoint accuracy and minimal cost.

### Partial motor preservation after SCI

Markers placed on either the fingers, wrist, elbow or acromion revealed preserved trajectories after C5 to C8 SCI during both reaching and reach-to-grasp [24, 25, 28, 35], attesting to the fact that SCI participants can produce efficient smooth trajectories similar to controls during reaching [25, 35] and reaching-to-grasp [35]. TPV and the shape of the velocity profiles [3, 26, 27] along with reaching accuracy (attesting to endpoint accuracy [3, 24, 25, 27]) were comparable with controls during reaching but not during reach-to-grasp and overhead movements. Indeed, motor control after SCI is characterized by strong kinematic invariants like movement endpoint accuracy and movement cost reduction as attested by the smooth trajectories and bell-shaped velocity profiles [45]. The relative preservation of these kinematic features is due to compensatory mechanisms, in particular during grasping and overhead movements as developed below.

### Shoulder and elbow coordination after SCI

Notwithstanding the overall reduction of degrees of freedom for the upper limb, SCI individuals show patterns of shoulder and elbow coordination similar to healthy individuals during reaching and reach-to-grasp [14, 24, 25, 39]. That is, the linear temporal relationship [24, 35] and the velocity coupling [36] between these joints are preserved, but at the cost of other reorganizational changes. The main change occurs at the scapulothoracic joint, where scapula rotation increases during reaching [35] and reach-to-grasp [15, 35], and both upward and external scapula rotations increase [15, 40] in order to orient and stretch out the upper limb [24]. A complementary change occurs at the glenohumeral joint, where increased abduction mitigates the reduced flexion during overhead movements [17]. These scapulothoracic and glenohumeral joint compensatory movements ensure that mechanical dynamic interactions between the shoulder and elbow produce elbow extension despite *triceps brachii* paralysis [24].

### Reduction of the upper limb workspace after SCI

SCI leads to extensive sensorimotor deficits, for example, strength loss due to partial innervation (see Table 1) which leads to a decrease in full shoulder active range of motion (i.e. reduced shoulder flexion and abduction angles [17, 38, 41]) and a reduction of scapula lateral rotation [41]. Several complementary hypotheses have been put forward to account for the reduction of the superior and superiomedial reaching workspace in C5 and C6 SCI individuals. One idea is that a strength deficit in the glenohumeral joint agonist could perturb these movements. Indeed, partial denervation of the *pectoralis major* (normal innervation C5-T1) results in decreased strength of the primary humeral adductor [26]. Increased EMG contraction intensity after SCI [38] might be a marker of the difficulty associated with these movements. A second idea is that a proximal deficit in *serratus anterior* (normal innervation C5-C7) means that this muscle cannot resist against the tension of the *rhomboids* antagonist muscles (normal innervation C4-C5) which results in both a reduction in scapula lateral rotation and in scapulothoracic instability. This could decrease the shoulder’s full active range of motion [41]. A third idea is that a distal deficit of the *triceps brachii* (normal innervation C7-T1) [26] might force C5-C6 SCI individuals to reduce overhead workspace in order to keep the elbow extended and to maintain the mechanical dynamic interaction between the shoulder and elbow. Fourth, shoulder ankylosis could reduce superiomedial and superior reach by reducting passive full range of motion by about 25° during both shoulder flexion and abduction compared with healthy individuals [38, 44]. A final factor is shoulder pain, which is reported by more than half of the individuals with tetraplegia [46], and probably contributes to the functional limitation of the workspace.

### Main temporal kinematic difference after SCI: prolonged movement time

The main consequence of SCI is increased MT [3, 14, 17, 25, 28, 35] which is associated with decreased PV [3, 14, 24, 28, 29, 35]. Acceleration and deceleration peaks are lower during reaching [25] and shoulder velocity decreases during overhead movements [17]. Motor slowing might be due to shoulder and elbow muscle strength decreases [28, 38], as SCI participants with weaker preserved upper limb muscles exhibit lower velocity and acceleration peaks and increased MTs. Importantly, motor slowing could also be due to *triceps brachii* paralysis which prevents agonist–antagonist co-contraction, which is observed in healthy individuals and serves to stop movement [25, 43]. Indeed, elbow flexion velocity decreases when *triceps brachii* is paralyzed but can be increased when a manipulandum provides a constant extensor torque [28]. Moreover, elbow stiffness due to impairment of agonist–antagonist co-contraction probably contributes to the alteration of reaching movements [24]. According to Fitt’s law and the speed-accuracy trade-off principle, motor slowing could reflect central adaptation to maintain accuracy at levels similar to those of healthy individuals [47]. This is consistent with the observation of reduced accuracy during fast elbow flexion movements [28]. Furthermore, grasping is the main cause of increased MT [29], as MT is nearly twice that of controls during grasping but only one and a half times longer during reaching and overhead movements (see Table 3). It is noteworthy that the reduction of MT is not related to the need to avoid trunk imbalance since trunk support did not increase velocity during fast upper limb movements [34].

### Requirements of grasping

#### Transport phase

Jeannerod described the transport and grasping phases of reach-to-grasp [48] and studies of the former phase classically used reaching movements. SCI and healthy individuals exhibit similar reaching movements, which favors the partial motor compensation previously discussed. Kinematic differences appear during grasping, however, with prolonged deceleration of the transport phase as attested by (i) delayed TPV [3, 14, 37], (ii) asymmetric velocity profile of both the hand and fingers [3, 35], and (iii) stable shoulder flexion, elbow extension and wrist tangential velocity before grasping [15]. These differences reflect the modification of the duration of the transport and grasping phases as the transport phase is initially quick but slows down towards the end [48]. Indeed, after SCI the transport phase of reach-to-grasp is characterized by reduced acceleration and prolonged deceleration durations, favoring final hand adjustment and successful grip. Finally, maximal grip aperture, a sign of grip preparation during transport [48], is lost because of finger extensor paralysis. Since grip aperture is passive and relies on wrist flexion and elbow pronation (favoring finger opening with gravity), grip preparation is effective only if the size of the object to be grasped is less than the maximal achievable grip width.

#### Grasping phase: tenodesis grasp

Wrist extension is a key-component of tenodesis grasp [3, 14, 15, 29]. Indeed, wrist extension and tendon shortening [3] lead to two passive grips: whole hand and lateral grip. In SCI participants wrist extension is greater during lateral than whole hand grip, suggesting a greater need for tendon shortening during lateral grip [3]. Increased hand height during reach-to-grasp brings the hand above the object thus preventing collision, and could explain EMG contraction intensity increases [15]. Joint coupling during tenodesis grasping is different from that observed in healthy individuals. Specifically, SCI individuals plan transport and grasping phases consecutively and the second velocity peak during grasping demonstrate a second movement following reaching [29, 35]. The negative correlation between the number of velocity peaks and the ASIA motor index indicates that the higher the SCI level the more fragmented the movement [37]. Finally, reaching and grasping are successively planned after C6-C7 SCI as attested by the wrist flexion during transport but the wrist extension during grasping [3, 14, 15, 29], and the loss of temporal coupling between the shoulder and wrist during reach-to-grasp [15]. This contrasts with normal reach-to-grasp where the grip is prepared during the transport phase [48].

#### Effect of elbow extension restoration

Restoration of elbow extension, either by tendon transfer surgery from *posterior deltoid* to *triceps brachii*[17, 26] or the use of a manipulandum that provides an extensor torque [28], decreases MT [17, 26, 28] and reduces errors [28]. This is due to the restoration of elbow stiffness which in turn decreases the need for glenohumeral compensatory movements during overhead movements. Specifically, the shoulder abduction angle decreases favoring flexion [17] while the shoulder joint velocity concomitantly increases [17]. Surgery also improves strength and general performance, and participants report high satisfaction after their operations [49]. Although functional improvements are reported, surgery fails to completely restore motor function [50] as shown by the absence of improvements during both superior and medial maximal reaching [26].

## Conclusion

SCI participants efficiently execute a broad range of upper limb tasks. Kinematic evidence shows that, even after SCI, movements are planned and executed according to strong kinematic invariants like movement endpoint accuracy and minimal cost. Elbow extension with a weak or paralyzed *triceps brachii* relies on increased movements of the scapulothoracic and glenohumeral joints. This provides a dynamic coupling between the shoulder and elbow which palliates the *triceps brachii* paralysis but limits the superior maximal reach. Moreover, reach-to-grasp planning is modified with reaching and grasping performed successively contrary to normal grasping patterns where grasping is prepared during the transport phase. The prolonged transport phase ensures correct hand placement while the grasping relies on wrist extension eliciting either whole hand or lateral grip. One main kinematic characteristic after tetraplegia is motor slowing attested by increased MT which can be a direct consequence of the strength deficit or due to a behavioral adaptation in order to preserve the accuracy of the movement and ensure grasping by tenodesis. Another issue concerns the reduction of maximal superior reaching during overhead movements. This can be caused by a strength deficit of either shoulder agonist or synergic muscles along with shoulder ankylosis and pain. Moreover, increased shoulder movements could favor shoulder pain, especially since this joint is already under severe strain. This could promote shoulder overuse pathologies which lead to pain and ankylosis, which then further contribute to the impairment. Indeed, further studies on open chain movements are needed to identify the contribution of each of these previous reasons in order to tailor upper limb rehabilitation programs for SCI individuals.

## Abbreviations

SCI: Spinal cord injury
C5/6/7/8 and T1/2: Cervical and thoracic vertebra indicating the level of spinal cord injury
ASIA: American Spinal Injury Association
AIS: ASIA Impairment Scale
US: United States of America
MT: Movement time
PV: Peak velocity
TPV: Time to peak velocity
EMG: Electromyogram
Nm: Newton per meter.
